# Risperidone-Induced Paradoxical Agitation in a Child With Lamb–Shaffer Syndrome, Autism Spectrum Disorder, and Attention Deficit Hyperactivity Disorder: A Case Report

**DOI:** 10.7759/cureus.99097

**Published:** 2025-12-13

**Authors:** Mohammed J Hayyan, Shereen J Alsaleem, Abdulraheem A Almubarak, Murtadha A Al Nas

**Affiliations:** 1 Department of Psychiatry, Prince Mohammed Bin Fahd Hospital, Qatif, SAU; 2 Department of Pediatrics, Prince Mohammed Bin Fahd Hospital, Qatif, SAU; 3 Department of Psychiatry, Qatif Central Hospital, Qatif, SAU

**Keywords:** agitation, aripiprazole, autism spectrum disorder, intellectual disability, lamb-shaffer syndrome, paradoxical reaction, risperidone, sox5

## Abstract

Lamb-Shaffer syndrome (LAMSHF) is a rare autosomal dominant neurodevelopmental disorder caused by haploinsufficiency of the SOX5 gene on chromosome 12p12.1 and characterized by global developmental delay, intellectual disability, speech and language impairment, and frequent behavioral disturbances, including autistic traits and attention deficits. Data on psychotropic response profiles in Lamb-Shaffer syndrome are extremely limited.

We report a six-year-five-month-old boy with de novo Lamb-Shaffer syndrome, autism spectrum disorder (ASD) of mild-to-moderate severity, attention deficit hyperactivity disorder, and query intellectual disability who developed paradoxical behavioral activation after initiation of very low-dose risperidone oral solution (0.12 mg once daily) for mild-to-moderate irritability, aggression, and self-injurious head banging. Shortly after treatment initiation, he exhibited marked agitation, increased hyperactivity, worsening aggression and self-injury, insomnia, restlessness, amplification of stereotypic movements, emotional lability, and prolonged crying episodes, clearly exceeding his baseline behavioral pattern and temporally associated with dosing. Clinical assessment and available laboratory investigations, including complete blood counts, liver and renal function tests, electrolytes, thyroid function, bone profile, vitamin B12, vitamin D, lactate, and ammonia, did not reveal metabolic, endocrine, or infectious etiologies. Risperidone was discontinued with subsequent resolution of the paradoxical symptoms and return to baseline. He was then switched to aripiprazole (2 mg once daily for 10 days, then 5 mg once daily), with gradual moderate-to-marked improvement in irritability and self-injurious behavior and no recurrence of agitation over several months of follow-up.

To our knowledge, this is the first reported case of risperidone-induced paradoxical activation in a child with Lamb-Shaffer syndrome. This case highlights the need for cautious antipsychotic use and close monitoring in rare genetic neurodevelopmental disorders and suggests that aripiprazole may represent a viable alternative when risperidone is poorly tolerated.

## Introduction

Lamb-Shaffer syndrome (LAMSHF, OMIM 616803) is a rare autosomal dominant neurodevelopmental disorder caused by heterozygous loss-of-function variants or microdeletions involving the SOX5 gene at chromosome 12p12.1 [1-3]. Clinical characterization from recent case series demonstrates a consistent phenotype of global developmental delay, mild-to-moderate intellectual disability, prominent expressive language impairment, mild facial dysmorphism, and behavioral disturbances, including autistic features, hyperactivity, and emotional dysregulation [1-3]. The phenotypic spectrum continues to expand, with reports of variable growth parameters, motor delay, and differences in severity between individuals even within the same family [1-3].

SOX5 encodes a transcription factor critical for corticogenesis and the development of cortical and subcortical projection neurons, providing a plausible mechanistic link between gene disruption and the observed neurodevelopmental and behavioral phenotype [1-3]. Despite growing knowledge of the clinical and molecular aspects of Lamb-Shaffer syndrome, little is known about psychopharmacologic response profiles or psychotropic tolerability in affected individuals.

Risperidone, a dopamine D₂ and serotonin 5-HT₂A receptor antagonist, is among the most widely used atypical antipsychotics in children for the treatment of irritability, aggression, and self-injurious behavior in autism spectrum disorder. Randomized controlled trials have demonstrated significant reductions in tantrums, aggression, and self-injury, with sustained benefits over time [4]. However, real-world studies in pediatric populations report a high burden of adverse events, including weight gain, metabolic abnormalities, hyperprolactinemia, and extrapyramidal symptoms [5].

Evidence for risperidone in individuals with ADHD and intellectual disability is more limited and often extrapolated from autism and disruptive behavior disorder studies [6]. In addition, a comparative study in children under six years of age reported that both risperidone and aripiprazole were effective in reducing ADHD symptoms, although the adverse event profiles differed between the two agents [7].

Paradoxical behavioral activation, defined as worsening agitation, irritability, or aggression following introduction of an antipsychotic, has been reported with risperidone and other atypical antipsychotics in adults with dementia, where some patients become more agitated and only improve after dose reduction or discontinuation [8].

Such reactions are insufficiently characterized in children, and virtually no data exist for rare monogenic neurodevelopmental disorders such as Lamb-Shaffer syndrome.

We report a child with de novo SOX5-related Lamb-Shaffer syndrome, ASD, ADHD, and queried intellectual disability who developed a paradoxical reaction to very low-dose risperidone, followed by clinical stabilization and improvement upon shifting to aripiprazole. This case underscores the need for gene-aware, cautious psychopharmacology in rare neurodevelopmental syndromes.

## Case presentation

Clinical history and developmental profile

A six-year-five-month-old boy was followed in pediatric genetics, metabolic, neurology, orthopedic, and psychiatry clinics for global developmental delay, failure to thrive, and behavioral dysregulation. At the time of the index psychiatric visit, his weight was 13.8 kg and his height 115 cm, both below the third percentile for age.

He was born at term by spontaneous vaginal delivery after an otherwise uneventful pregnancy to non-consanguineous but distantly related parents. There were no reported antenatal complications, perinatal asphyxia, or neonatal sepsis, and he was discharged home with his mother in good condition.

Developmental delays became evident in infancy. He sat independently at approximately nine months, stood with support at 21 months, and walked independently at around 22 months, with an abnormal gait and out-toeing. Hypotonia and poor weight gain were documented in early childhood.

Expressive language development was markedly delayed, with only a few simple words such as “mama” and “baba” and no progression to age-appropriate phrase speech. Multiple evaluations by speech and language therapists described severe language delay, limited functional vocabulary, poor sitting tolerance, hyperactivity, and difficulty following commands.

He relied heavily on nonverbal communication, including gestures and physical guidance, to express needs. Socially, he showed reduced eye contact, limited reciprocal interaction, and a preference for solitary or screen-based activities, including watching repetitive video clips and observing moving air currents.

Family history

The parents are distantly related. One older sister has been diagnosed with a complex chromosomal disorder consistent with Cri-du-chat syndrome and multiple copy number changes involving chromosomes 2p, 3p, and 5p; she exhibits significant developmental delay and dysmorphic features.

Another brother is clinically well, with typical development and no neurodevelopmental concerns reported, and has not undergone genetic testing to date. A broader family history of suspected autism spectrum traits and developmental delay was reported among paternal male relatives, although no formal diagnoses had been documented.

Medical, neurologic, and laboratory evaluation

He had no previous surgeries apart from extensive dental rehabilitation performed under general anesthesia. Brain MRI was reported as normal, without structural malformations or white matter abnormalities. Audiology and ophthalmologic assessments did not reveal hearing or visual deficits sufficient to account for his global developmental delay.

Cardiology evaluation, including electrocardiogram (ECG) and echocardiogram, showed a structurally normal heart with preserved biventricular function and only trivial physiologic valvular regurgitation.

Serial laboratory investigations included complete blood counts, liver and renal function tests, serum electrolytes, bone and mineral profiles, thyroid function studies, vitamin B12, ferritin, vitamin D, lactate, and ammonia. Overall, results were within or close to reference ranges for age, with mild microcytic anemia and low ferritin consistent with iron deficiency and mildly suboptimal vitamin D levels. There were no findings suggestive of an inborn error of metabolism, systemic inflammatory process, or endocrine disorder.

Genetic findings

Given persistent global developmental delay and failure to thrive despite normal neuroimaging and metabolic workup, he was referred to a tertiary genetics center. Cytogenetic studies revealed a balanced translocation in the father, described as 46,XY(2;5)(p23;p15.3), and a normal karyotype in the mother. Molecular testing in the proband identified a pathogenic alteration involving chromosome 12p12.1 consistent with SOX5 haploinsufficiency and autosomal dominant Lamb-Shaffer syndrome. His older sister showed complex chromosomal copy number variations; his phenotypically normal brother had not yet been tested.

Neurodevelopmental and behavioral-psychological assessment

Adaptive functioning, as reported by caregivers on standardized adaptive behavior questionnaires, was in the low range, consistent with moderate impairment. Attempts to quantify intellectual function using a nonverbal IQ test were unsuccessful due to poor cooperation, prominent repetitive behaviors, and difficulty sustaining attention and following instructions. Therefore, he was clinically conceptualized as having a query intellectual disability, with the severity not formally quantified.

Autism spectrum disorder was diagnosed by a child and adolescent psychiatrist and a clinical psychologist following a comprehensive assessment that integrated detailed developmental history, direct observation of the child in different settings, and caregiver reports. The overall presentation was judged to be in the mild-to-moderate range, with reduced eye contact, limited social reciprocity, restricted interests, sensory-seeking behaviors, and stereotypies such as repetitive finger movements and circling.

Standardized parent- and teacher-completed ADHD rating scales indicated clinically significant inattention and hyperactivity/impulsivity across settings, consistent with attention-deficit/hyperactivity disorder and associated with emotional and behavioral dysregulation.

Functionally, he remained dependent on caregivers for dressing, washing, and toileting and was not toilet-trained. He attended a special educational setting and received physiotherapy, speech therapy, and occupational therapy.

Baseline psychiatric presentation

By the age of six years, his parents reported recurrent episodes of mild-to-moderate irritability, tantrums, and self-injurious behavior, particularly repetitive head banging when he was frustrated, denied preferred items, or when routines were disrupted. These episodes occurred on multiple days each week, were difficult to redirect despite consistent behavioral strategies, and posed a risk of physical injury and significant stress for the family.

Despite structured routines and ongoing non-pharmacologic interventions, including psychological, speech, and occupational therapy, these behaviors persisted and continued to interfere with his daily functioning, prompting the need for psychiatric evaluation and intervention.

At psychiatric review, he appeared small for age, with poor eye contact, high motor activity, limited comprehensible speech, and prominent stereotypic movements. Affect was labile, with rapid shifts from apparent enjoyment to frustration and crying. No hallucinations, delusions, or other psychotic features were evident; mood was difficult to assess due to limited language. At that time, he had not previously received psychotropic medications.

In light of the persistence of irritability and self-injury, the associated risk of harm, and the limited response to non-pharmacologic interventions, a decision was made to initiate a cautious trial of low-dose risperidone oral solution targeting irritability and aggression.

Risperidone initiation and paradoxical reaction

Risperidone oral solution (1 mg/mL) was initiated at a dose of 0.12 mL once daily (0.12 mg/day; approximately 0.01 mg/kg/day), administered in the evening. This very low starting dose was chosen in view of his low body weight, underlying neurodevelopmental vulnerability, and the presence of a rare monogenic syndrome. At that time, no other psychotropic medications were started or adjusted.

Within a few hours of the first dose, his parents noted a clear change in behavior compared with his usual baseline. He became markedly more agitated, with increased motor restlessness, pacing, and an inability to settle. Over the course of the first week of treatment, these symptoms intensified and generalized. In contrast to his prior intermittent, mild-to-moderate episodes, he now displayed frequent and severe agitation, more intense self-injurious behavior (including hitting his head with his hands and against the wall), escalating physical aggression, and throwing objects toward caregivers and in the environment.

He was described as crying and screaming for prolonged periods, with pronounced emotional lability and difficulty being consoled. Sleep became significantly disrupted, with delayed sleep onset, frequent nighttime awakenings, and reduced total sleep time. His appetite also declined noticeably over the same period. These changes were temporally related to risperidone dosing and were perceived by his caregivers and treating clinicians as qualitatively and quantitatively different from his established pre-treatment behavioral pattern.

During this period, there were no reported fevers, intercurrent infections, new pain behaviors, seizures, or major environmental changes at home or school. Physical examination remained unremarkable, and previously available laboratory studies did not suggest a metabolic, endocrine, or systemic inflammatory process. In the absence of an alternative medical explanation and given the clear temporal relationship, a paradoxical behavioral reaction to risperidone was suspected.

The family was instructed to taper and discontinue risperidone over the ensuing days. Following complete discontinuation, his agitation, aggression, and sleep disturbance gradually improved, and within several days, he returned to a level of behavior comparable to his pre-risperidone baseline, with resolution of the newly emergent severe agitation.

Switch to aripiprazole and follow-up

After discussing the reaction and available options with the family, aripiprazole was selected as an alternative atypical antipsychotic. Treatment was initiated at 2 mg once daily for 10 days, followed by an increase to 5 mg once daily, which he continued thereafter.

Over the subsequent weeks, his parents reported a gradual but clear improvement in behavior. The frequency and intensity of irritability and tantrums decreased, and self-injurious head banging became less frequent and less severe. He was better able to participate in daily routines and structured activities, and episodes of prolonged inconsolable crying were no longer reported. Sleep improved compared with the risperidone period, and his appetite returned toward baseline.

No extrapyramidal symptoms, acute dystonic reactions, excessive sedation, or clinically significant weight gain were observed on aripiprazole. With regular monitoring in the clinic, his behavior remained more stable, and there was no recurrence of the severe, pervasive agitation that had emerged during the risperidone trial.

## Discussion

This case describes a child with de novo SOX5-related Lamb-Shaffer syndrome, ASD, ADHD, and query intellectual disability who developed paradoxical behavioral activation after initiation of very low-dose risperidone, with resolution after discontinuation and subsequent benefit from aripiprazole. To our knowledge, this is the first report to specifically highlight a paradoxical risperidone reaction in Lamb-Shaffer syndrome.

Lamb-Shaffer syndrome and behavioral phenotype

Recent clinical series have delineated Lamb-Shaffer syndrome as a distinctive SOX5-related neurodevelopmental disorder characterized by developmental delay, intellectual disability, expressive language impairment, mild facial dysmorphism, and a high prevalence of behavioral difficulties, including autistic traits and attentional problems [1-3]. Our patient’s phenotype, motor and language delay, low adaptive functioning, ASD features, and behavioral dysregulation are consistent with these descriptions.

Despite this, psychopharmacologic data in Lamb-Shaffer syndrome are almost nonexistent. The published case series rarely describe specific psychotropic agents or response patterns [1-3]. As more individuals with SOX5 variants are identified, understanding medication tolerability and response will become increasingly important for clinical care.

Risperidone, autism, and intellectual disability

Risperidone has strong evidence for treating irritability, aggression, and self-injurious behavior in children with autism spectrum disorder, leading to its regulatory approval for this indication [4]. However, observational studies demonstrate that a large proportion of pediatric patients experience at least one adverse event, particularly weight gain and metabolic changes, as well as extrapyramidal symptoms [5].

Evidence for risperidone in individuals with ADHD and intellectual disability is limited; a Cochrane-type review noted the absence of randomized trials specifically targeting ADHD in people with intellectual disability and highlighted that current practice largely extrapolates from autism and disruptive behavior literature [6]. Thus, the decision to use risperidone in this case was grounded in its established efficacy in ASD-related irritability but extrapolated to a child with a rare genetic syndrome and probable intellectual disability, after non-pharmacologic interventions had provided only partial benefit.

Paradoxical antipsychotic reactions

Paradoxical worsening of agitation and behavioral disturbance has been described with risperidone and other atypical antipsychotics in adults with dementia, where some patients demonstrate increased agitation that improves only after dose reduction or discontinuation [8]. Proposed mechanisms include medication-induced akathisia, unmasking of underlying behavioral disinhibition, or idiosyncratic hypersensitivity of dopaminergic circuits in a vulnerable brain.

In pediatric populations, data on paradoxical antipsychotic activation are sparse and largely anecdotal. The pediatric risperidone safety study identified self-injurious behavior as a predictor of adverse events in general but did not systematically characterize paradoxical behavioral activation [5]. Our patient had clinically significant but intermittent self-injurious head banging at baseline, which may reflect a particularly vulnerable neurobehavioral phenotype. However, the clinical picture that emerged after risperidone initiation was qualitatively and quantitatively different, characterized by pervasive severe agitation, markedly intensified self-injurious head banging (including hitting his head with his hands and against the wall), increased physical aggression with throwing objects, severe insomnia with frequent nighttime awakenings, and prolonged inconsolable crying episodes.

In this case, several features support a paradoxical causal reaction to risperidone: 1) A clear and reproducible temporal association between risperidone initiation and behavioral deterioration. 2) Qualitative and quantitative changes beyond his established baseline patterns, including new-onset severe insomnia and global agitation. 3) Absence of alternative medical or environmental explanations on clinical assessment and laboratory evaluation. 4) Resolution of symptoms after discontinuation of risperidone. 5) Subsequent tolerance and benefit with a different atypical antipsychotic (aripiprazole).

Notably, the dose used (0.12 mg/day, approximately 0.01 mg/kg/day) was far lower than standard pediatric dosing ranges, suggesting heightened pharmacodynamic sensitivity rather than simple dose-related toxicity.

Possible role of SOX5-related brain vulnerability

SOX5 plays an essential role in corticogenesis and neuronal differentiation; SOX5 haploinsufficiency has been shown to disrupt cortical projection neuron development and connectivity [1-3]. It is plausible that such structural and circuit-level vulnerabilities alter dopaminergic and serotonergic signaling and thereby modulate sensitivity to pharmacologic agents acting on these systems. In rare monogenic neurodevelopmental disorders, individual variability in receptor density, network stability, and neural excitability may predispose to atypical or exaggerated responses to psychotropics.

Although speculative, this case raises the possibility that children with Lamb-Shaffer syndrome may be at increased risk of paradoxical reactions to certain antipsychotics. Systematic collection of psychopharmacologic data in larger SOX5 cohorts is needed to investigate this hypothesis.

Aripiprazole as an alternative

Aripiprazole, a partial dopamine D₂ and serotonin 5-HT₁A agonist and 5-HT₂A antagonist, is also approved for the treatment of irritability in children and adolescents with autism. Comparisons between risperidone and aripiprazole in young children suggest both agents can be effective, with differences in onsets of action and side-effect profiles [7]. In a study of children under six years old, both medications improved ADHD symptoms, but adverse event profiles varied [7].

In our patient, aripiprazole was associated with gradual improvement in irritability and self-injury and no recurrence of paradoxical agitation, supporting its potential usefulness in cases where risperidone is poorly tolerated. Whether the partial agonist properties of aripiprazole confer advantages in SOX5-related neurodevelopmental disorders remains unknown, but this case suggests that switching to an alternative agent with a different pharmacodynamic profile is a reasonable strategy when paradoxical reactions occur.

## Conclusions

This case describes a child with Lamb-Shaffer syndrome who developed a rapid and significant paradoxical behavioral activation following very low-dose risperidone, with complete resolution after discontinuation and sustained behavioral improvement on aripiprazole. While conclusions from a single case must remain cautious, the temporal pattern strongly suggests an atypical medication response rather than a natural fluctuation in baseline behavior. This observation highlights the possibility that individuals with rare neurodevelopmental syndromes, such as SOX5 haploinsufficiency, may demonstrate heightened sensitivity to certain antipsychotics.

The findings underscore the importance of careful dosing, slow titration, and vigilant monitoring when initiating risperidone or other antipsychotics in similar populations. This case contributes to hypothesis generation regarding psychopharmacologic variability in monogenic neurodevelopmental disorders and emphasizes the need for systematic data collection to better define safety, responsiveness, and alternative treatment strategies in Lamb-Shaffer syndrome.

## References

[1] Zhu GQ, Dong P, Li DY (2025). Clinical characterization of Lamb-Shaffer syndrome: a case report and literature review. BMC Med Genomics.

[2] Lian R, Wu G, Xu F (2025). Clinical cases series and pathogenesis of Lamb-Shaffer syndrome in China. Orphanet J Rare Dis.

[3] Edgerley K, Bryson L, Hanington L (2025). SOX5: Lamb-Shaffer syndrome-A case series further expanding the phenotypic spectrum. Am J Med Genet A.

[4] McDougle CJ, Scahill L, Aman MG (2025). Risperidone for the core symptom domains of autism: results from the study by the autism network of the research units on pediatric psychopharmacology. Am J Psychiatry.

[5] Oshikoya KA, Carroll R, Aka I (2025). Adverse events associated with risperidone use in pediatric patients: a retrospective biobank study. Drugs Real World Outcomes.

[6] Thomson A, Maltezos S, Paliokosta E, Xenitidis K (2025). Risperidone for attention-deficit hyperactivity disorder in people with intellectual disabilities. Cochrane Database Syst Rev.

[7] Yousefsani BS, Moharreri F, Baniasadi M, Marvasti F (2025). A comparative study of risperidone and aripiprazole in attention deficit hyperactivity disorder in children under six years old: a randomized double-blind study. Iran J Pediatr.

[8] Florea T, Palimariciuc M, Chirita V, Chirita R (2025). Paradoxical side effect in treatment with antipsychotics for aggression/agitation and psychosis in dementia: case-series. Bull Integr Psychiatry.

